# Early warning systems for malaria outbreaks in Thailand: an anomaly detection approach

**DOI:** 10.1186/s12936-024-04837-x

**Published:** 2024-01-08

**Authors:** Oraya Srimokla, Wirichada Pan-Ngum, Amnat Khamsiriwatchara, Chantana Padungtod, Rungrawee Tipmontree, Noppon Choosri, Sompob Saralamba

**Affiliations:** 1 Nuffield Department of Medicine, University of Oxford, Broad St, Oxford, OX13AZ UK; 2 Mahidol Oxford Tropical Medicine Research Unit, Faculty of Tropical Medicine, Mahidol University, Ratchawithi Rd, Bangkok, 10400 Thailand; 3 Department of Tropical Hygiene, Faculty of Tropical Medicine, Mahidol University, Ratchawithi Rd, Bangkok, 10400 Thailand; 4 Center of Excellence for Biomedical and Public Health Informatics, Faculty of Tropical Medicine, Mahidol University, Ratchawithi Rd, Bangkok, 10400 Thailand; 5 Division of Vector Borne Diseases, Department of Disease Control, Ministry of Public Health, Talat Kwan, Bangkok, Nonthaburi, 11000 Thailand; 6 College of Arts, Media and Technology, Chiang Mai University, Sukhothai 5 Alley, Mueang Chiang Mai, Chiang Mai, 50200 Thailand

**Keywords:** Malaria, Early detection, Outbreak, Anomaly detection

## Abstract

**Background:**

Malaria continues to pose a significant health threat. Rapid identification of malaria infections and the deployment of active surveillance tools are crucial for achieving malaria elimination in regions where malaria is endemic, such as certain areas of Thailand. In this study, an anomaly detection system is introduced as an early warning mechanism for potential malaria outbreaks in countries like Thailand.

**Methods:**

Unsupervised clustering-based, and time series-based anomaly detection algorithms are developed and compared to identify abnormal malaria activity in Thailand. Additionally, a user interface tailored for anomaly detection is designed, enabling the Thai malaria surveillance team to utilize these algorithms and visualize regions exhibiting unusual malaria patterns.

**Results:**

Nine distinct anomaly detection algorithms we developed. Their efficacy in pinpointing verified outbreaks was assessed using malaria case data from Thailand spanning 2012 to 2022. The historical average threshold-based anomaly detection method triggered three times fewer alerts, while correctly identifying the same number of verified outbreaks when compared to the current method used in Thailand. A limitation of this analysis is the small number of verified outbreaks; further consultation with the Division of Vector Borne Disease could help identify more verified outbreaks. The developed dashboard, designed specifically for anomaly detection, allows disease surveillance professionals to easily identify and visualize unusual malaria activity at a provincial level across Thailand.

**Conclusion:**

An enhanced early warning system is proposed to bolster malaria elimination efforts for countries with a similar malaria profile to Thailand. The developed anomaly detection algorithms, after thorough comparison, have been optimized for integration with the current malaria surveillance infrastructure. An anomaly detection dashboard for Thailand is built and supports early detection of abnormal malaria activity. In summary, the proposed early warning system enhances the identification process for provinces at risk of outbreaks and offers easy integration with Thailand’s established malaria surveillance framework.

**Supplementary Information:**

The online version contains supplementary material available at 10.1186/s12936-024-04837-x.

## Background

Malaria remains a life-threatening and preventable disease in many parts of the world [1]. While significant progress in reducing Thai malaria cases has occurred in the past two decades, continued efforts are necessary to achieve elimination [2, 3]. The Operational Plan 2017–2021, aligned with Thailand’s National Malaria Elimination Strategy 2017–2026, emphasizes the need to enhance rapid identification of infections and implement timely and active surveillance and response measures to prevent further transmission [4]. The Division of Vector-Borne Disease (DVBD) leads the national malaria programme and is responsible for implementing surveillance initiatives in Thailand [3]. The DVBD, operating under the Department of Disease Control of the Ministry of Public Health, facilitated and oversees real-time aggregation of electronic malaria case data [4, 5]. The electronic malaria information system (eMIS) was developed by the Center of Excellence for Biomedical and Public Health Informatics (BIOPHICS), Faculty of Tropical Medicine at Mahidol University aiming to replace paper-based malaria reporting with near-real-time electronic reporting [5]. BIOPHICS currently hosts all eMIS data, acting as the ongoing technical system support for the ministry [5].

With the development of eMIS, Thailand has conducted the 1-3-7 strategy to improve malaria elimination [6]. This strategy involves notifying each malaria case within 1 day of testing positive for malaria, classifying the case within 3 days, and completing a response within 7 days [6]. Responses involve case investigation and the deployment of appropriate interventions for vector control [4]. Depending on the case classification, interventions include blood sampling, distributing insecticide-treated bed nets, indoor residual spraying, and health education [4]. Seasonal malaria chemoprevention (SMC) has been used as preventative treatment in areas with seasonal transmission and require adaptable thresholds to define disease patterns over time and space. These thresholds are used as a surveillance method to identify suitable areas for SMC and require input from health districts as they are often challenging to define [7]. Overall, these methods require a broad workforce, high data quality, continued leadership, and are costly. To successfully eliminate malaria in Thailand, it is crucial to incorporate well supported community-based health workers and establish an affordable and efficient detection system. This system should quickly identify outbreaks in their early stages, be adaptable to various health districts’ needs, and reduce the malaria burden more rapidly in the remaining localized high transmission foci [8].

Early warning systems give advance warnings of impending epidemics and play a crucial role in the malaria surveillance program overseen by the DVBD [9, 10]. Malaria outbreaks are defined as higher than usual malaria case activity in a specific area. Currently, the warning system relies on a 3 year median approach, where an alert is triggered if weekly malaria cases exceed the 3 year median of weekly cases from previous years and prompt investigation by the DVBD [11]. The publicly available online dashboard for the Thailand Malaria Elimination Programme (https://malaria.ddc.moph.go.th/malariaR10/index_newversion.php), provides information on provincial case counts, weekly case counts, 3 year median thresholds, and the implementation of the 1-3-7 strategy throughout Thailand. This tool provides general case visualization across Thailand, but it does not support identification of unusual malaria case activity across Thailand [11]. The development of improved early warning mechanisms and a robust dashboard is needed to optimize the response time and allocation of resources to areas with impending epidemics and to support effective implementation of preventive measures.

Anomaly detection is used to discover unexpected or rate events in data streams and can be applied to health data to identify outliers in a system [12]. Anomaly detection algorithms are dynamic and can include a combination of statistical and machine learning approaches and threshold-based methods that detect highly abnormal activities in the data. Examples of uses are fraud detection in insurance and banking, intrusion detection of computer networks, and medical informatics for disorder detection [12]. Three types of machine learning-based anomaly detection algorithms are supervised, unsupervised, and semi-supervised [12]. While no single anomaly detection method is universally effective, several approaches are suited for time series anomaly detection. These approaches include predictive confidence levels, statistical profiling, clustering, and density-based profiling [12–16]. Anomaly detection presents a promising approach in disease detection. Previous studies have explored the application of density-based anomaly detection algorithms to health data including heart disease, diabetes, and hepatitis [17, 18].

Similarly, the use of unsupervised anomaly detection methods have been used to discover implausible electronic health records in cancer registries [19] and adverse health conditions for people living dementia using sensor-base data [20]. In a study exploring the use of unsupervised anomaly detection for disease surveillance, Brazilian Amazon malaria surveillance data is used as a case study for early detection of outbreaks [21]. As anomaly detection algorithms are a promising technique for early identification of abnormal malaria activity, the use of both unsupervised clustering and time series-based anomaly detection methods are explored for endemic malaria environments similar to Thailand. This study focuses on using anomaly detection algorithms as a method to strengthen malaria surveillance systems with Thailand as an example setting. The early detection of impending outbreaks can be integrated with the existing eMIS and enhance the current 1-3-7 strategy to effectively respond to any anomaly identification within 7 days using appropriate interventions [4].

The aim of this research is to propose an early detection system to support the malaria elimination programs in countries where malaria is endemic, similar to Thailand. Additionally, the aim is to improve methods for early detection of malaria in areas with impending outbreaks. To achieve these aims and using Thailand as an example setting, the main research objectives are:

1. Develop anomaly detection algorithms and early detection thresholds that are suitable for malaria data in Thailand.
2. Compare the developed algorithms to Thailand’s current early warning threshold.
3. Develop a prototype user interface for Thai public health professionals that supports early identification of outbreaks and enables focused attention on anomalous areas.

## Methods

To support the objectives of this study, the methods are separated into five main sections: data, algorithms, algorithm comparison, code structure, and user interface.

### Data

**Fig. 1:**
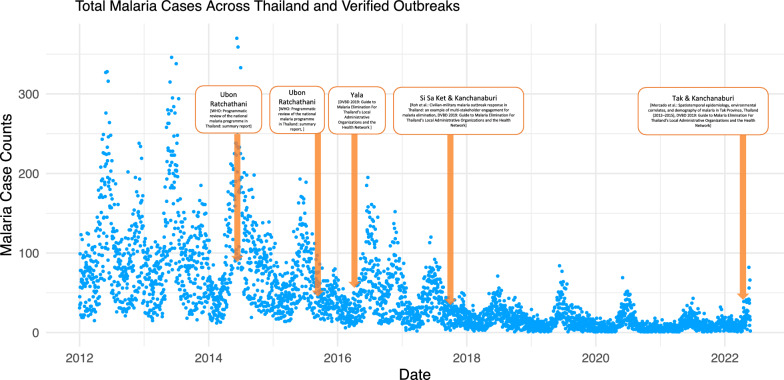
Total Malaria Cases Across Thailand from 2012 to 2022. The malaria case counts across Thailand are shown from 2012 to 2022. The verified outbreak dates, found in literature, are highlighted in orange and provide information on the province name and the reference used for each outbreak. These outbreak dates are used to compare and validate the anomaly detection algorithms presented in this paper

The data for this analysis was provided by the Ministry of Public Health and used under a research protocol approved by the Ethics Committee of the Faculty of Tropical Medicine, Mahidol University, Bangkok. This study encompasses all 77 provinces of Thailand (see Additional file 1), a region characterized by its warm, humid tropical climate and seasonal monsoon winds [22]. Thailand has an annual cycle of wet and dry seasons with a concentration of rainfall during the wet season [23]. Thailand is located in Southeast Asia and is bordered by Myanmar in the west, Laos in the north, Cambodia in the east, and Malaysia in the south [24]. The data contains Thailand malaria cases reported daily from 2012 to 2022 for all Thai provinces with personal identifiers excluded from the analyses. The data contains 31 variables including the blood draw date, nationality, sex, age, province, province ID, subdistrict, species of malaria, border type, occupation, and treatment for 180256 observations of malaria cases. All province names are translated into English based on their provincial ID (details in Additional file 1). The data is transformed into incidence data based on the case counts per date and then grouped based on province for further analysis. Depending on the method, the case data was aggregated either daily, weekly, or monthly (see Additional file 11 for more information about aggregation interval for each method). Initial visualization of the data is shown (see Fig. 1 and Additional file 2) and can be further visualized in the analytics tab of the final dashboard: https://moru.shinyapps.io/Malaria_Anomaly_Detection_App/.

As climate and environmental factors could be one of the primary factors driving malaria transmission [25–27], daily precipitation and temperature data is incorporated in an unsupervised clustering method with daily malaria cases. From both the temperature and precipitation datasets, measurements from central Thailand are used for analysis on all provinces as the temperature and precipitation does not vary significantly across Thailand. The daily precipitation data for Thailand was extracted from Temperature and Precipitation Gridded Data for Global and Regional Domains Derived from In-situ and Satellite Observations from the Copernicus Climate Data Store [28]. Similarly, the daily temperature data for Thailand was extracted from the Berkeley Earth’s Global Temperature Gridded Data [29]. The daily precipitation and temperature data are added to the daily malaria incidence data frame based on date and scaled to be comparable to the malaria case data. The multivariate scaled data is then used as an input into density-based unsupervised clustering function DBSCAN. Unsupervised clustering approaches are further described below and in Additional file 11.

### Anomaly detection algorithms

Two main types of anomaly detection algorithms developed are clustering-based methods and time series-based methods. Each method will be used to identify anomalous or unusual malaria activity. The performance for each type of anomaly detection algorithm is compared in the following section. Table 1 shows the methods used for this analysis.

**Table 1:** Methods used for anomaly detection and their references

Method	References
Statistical profiling	[12, 38–42]
Predictive confidence interval	[42–45]
Unsupervised clustering^1^	[12, 16, 37, 46, 47]
Weekly case comparison	[4] 06/01/2024 06:55:00
Monthly case comparison	[48]
Rolling historical average^2^	
Weekly 3 year median	[11]

^1^Two main unsupervised clustering techniques are used for this method: time-series clustering with tsclust [37] and density-based clustering using DBSCAN

^2^This method was developed based on a combination of techniques from statistical profiling and weekly case comparison

Unsupervised clustering approaches create measurements between different elements and cluster them base on their similarity without requiring training data [12]. Anomalous observations are labelled when they have a high distance to existing clusters or have a lower density when compare to other clusters [12]. Anomaly detection algorithms based on unsupervised clustering approaches include unsupervised time-series clustering, unsupervised density-base clustering with the malaria case data, and unsupervised density-based clustering with malaria case data, precipitation data, and temperature data (see Additional file 11 for detailed descriptions).

Time series-based anomaly detection approaches analyse the data based on a sliding window and at a specified time frame. These methods are able capture the change in malaria cases for an evolving time series and can involve the comparison of cumulative cases, mean cases, and standard deviation along the time series. Time series-based anomaly detection algorithms include statistical profiling, predictive confidence interval, weekly and monthly malaria case comparisons, rolling historical averages, and weekly 3 year median case comparisons (see Additional file 11 for detailed descriptions).

As an initial test, early detection methods are applied and visualized at a provincial level to see if unusual case activity can be identified using this dataset. All methods can be selected in the dropdown menu in the analytics tab of the final dashboard (https://moru.shinyapps.io/Malaria_Anomaly_Detection_App/) and are grouped by clustering-based (orange) and time series-based (blue) (see Fig. 3).

### Algorithm validation and comparison

To validate the algorithms, additional literature review, the online Thailand Malaria Elimination Program tool, and consultation with BIOPHICS provided information on dates and provinces where malaria outbreaks were previously reported. To match available malaria data, outbreaks reported from 2012 to 2022 were selected. The two main goals for the validation stage are to identify the number of outbreaks caught for each method up to two weeks prior to the verified outbreak date, and the number of alerts triggered by each method.

From literature, the Thailand Malaria Elimination Program online tool, and consultation with BIOPHICS, 7 outbreak dates were identified. 6 of the 7 outbreaks were reported at a provincial level while 1 (2017 Kanchanaburi) was reported at a subdistrict level. Reported outbreaks are generally clustered along provinces bordering Laos, Cambodia, and Myanmar and could have resulted from factors like migrant movement, limited access to malaria prevention and diagnostics, inadequate monitoring measures, dense forest regions, and political and social unrest [30]. The summary of outbreak dates are shown in Table 2 (see Additional file 12 for detailed descriptions).

**Table 2:** Outbreak dates reported in literature from 2012 and 2022

Date Reported	Province	Reference
2014	Ubon ratchathani	[49]
2015	Ubon ratchathani	[49]
2016	Yala	[50]
2017	Si Sa Ket	[48] 06/01/2024 06:55:00
2017	Kanchanaburi	[50]
2022	Kanchanaburi	[50]^1^
2022	Tak	[51]^1^

^1^Additional analysis of case data shows large spike and from consultation with BIOPHICS

All anomaly detection methods are run through all the provinces. Each province and method were assessed to determine if it could generate warnings within a two-week window leading up to the outbreak date. The exact outbreak date, shown as a peak in cases, is found using the Thailand Malaria Elimination Programme online tool and compared to estimates reported in literature. The function summed the total real outbreak dates each method caught and the total number of alerts each method produced. The pseudocode for validating and comparing various anomaly detection methods can be found in Additional file 13 and the final result from testing is shown in the analytics tab of the final dashboard.

In addition to reporting verified outbreaks, the total number of alerts reported from each method are also tracked. Each anomaly detection method is applied to all the malaria data from 2012 to 2022 and reports the number of anomalies or alerts each method triggers. The purpose of tracking these alerts is to ensure that the method used for anomaly detection is not highly sensitive to every irregularity found in the case data and reporting is done for only highly anomalous activity.

### Code structure

The code is structured to conduct anomaly analysis at a provincial level, with a user-defined method, time frame, and malaria species (see Additional file 10). The data is converted into incidence data based on the resolution of analysis and grouped at a provincial level. The resolution of analysis can be increased to smaller regions; however, this will be more computationally intensive as increasing the resolution to the subdistrict level will take 45 times as long to run. After the user-specified method is applied to each province, the daily anomalous activity is reported for the time frame defined and stored in an outer data frame. The final activity data frame is used for further analysis and is connected to visualizations in the user interface in the form of a map highlighting anomalous provinces.

### Interface

The user interface is designed for the DVBD surveillance team with consultation through BIOPHICS. For easy visualization and prototyping, a wireframe of the inter-face was developed using Canva [31]. An R Shiny application was developed to test and debug functions, integrating visualization tools like raster, rworldmap, and ggplot to highlight anomalous activities [32–35].

The final application was created using R Shiny and bs4Dash and has three main pages [36]. The first page describes the project and the algorithms available for analysis. The second page provides a weekly summary, including information on provinces with detected anomalies. The third page allows the user to conduct further analysis by inputting the time frame, method, and species of malaria used for analysis. Two main visualizations are updated every time a new analysis is initiated: one highlighting provinces with anomalies detected and another showing the standardized incidence ratio of malaria incidence across Thailand. Additional information such as trend lines, percentage of provinces with anomalies detected, and names of provinces with unusual activity are also included.

## Results

### Algorithm development and validation

**Fig. 2:**
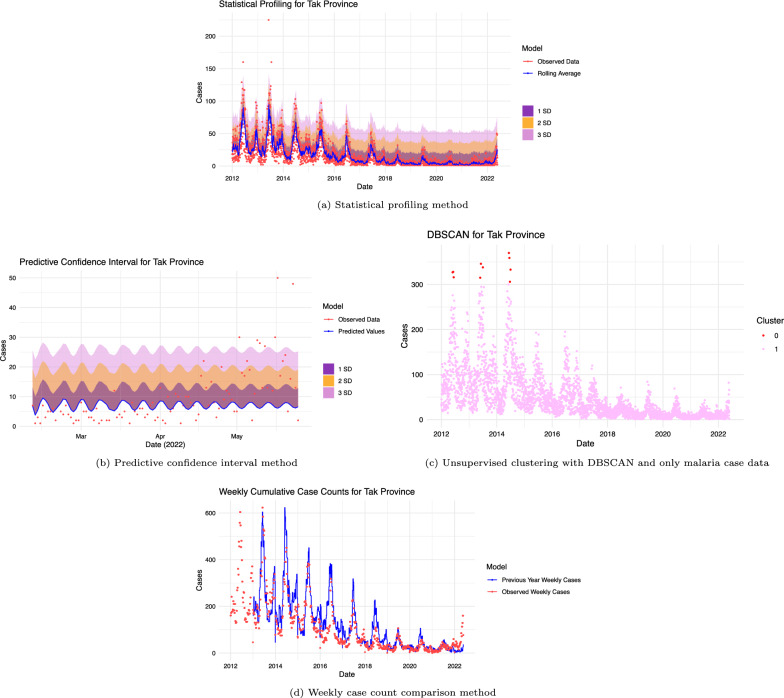
Visual results from testing algorithms with the Tak Province. Anomaly detection algorithms tested with Tak provincial malaria data from 2012 to 2022. **a** the statistical profiling method shows 3 standard deviation bands. Observations falling outside the 3 standard deviation bands are classified as anomalous. **b** the predictive confidence interval method is used to create 3 standard deviation bands from the mean standard error. Observations falling outside the 3 standard deviation band are classified as anomalous. **c** the unsupervised method using DBSCAN is used to cluster observations. Observations in cluster 0 (smallest cluster) are defined as anomalous while observations in cluster 1 are not. **d** the weekly cumulative case comparison method is used to compare observations. Weeks where cumulative cases are higher than the previous year’s weekly cases (blue) are classified as anomalous

A total of 9 anomaly detection algorithms were created and initially tested and visualized to confirm correctly implemented alerts were produced for observations exceeding thresholds or bands defining anomalous activity for the Tak province (see Fig. 2 and Additional file 3). From this initial test, anomalous observations are distinguished from normal malaria case activity.

After developing and validating the anomaly detection algorithms in the Tak province, tested each method was tested across all provinces to evaluate their effectiveness in identifying confirmed outbreaks. These results are shown in Table 3. In this table, the ✘ symbol shows that the method used did not trigger anomalous alerts at least two weeks before the verified outbreak and the ✔ symbol shows that the method used triggered anomalous alerts at least two weeks before this verified outbreak. The sensitivity for each method is calculated by taking the number of verified outbreaks found over the total number of verified outbreaks. In the analysis using time-series methods, varying levels of sensitivity was observed. Specifically, for the monthly case comparison, statistical profiling, and predictive confidence interval methods, the sensitivities were 0/7, 1/7, and 3/7, respectively. In contrast, the historical average, weekly case comparison, and weekly 3 year median methods demonstrated a higher sensitivity, each achieving a rate of 6/7. However, for the clustering-based methods, the sensitivity was consistently found to be 0/7. The highest number of verified anomalies found was 6 out of the 7. Methods able to identify 6 outbreaks were historical average, weekly case counts, and the weekly 3 year median method. Of these three methods, the historical average method produced the lowest number of alerts (see Additional file 4 for visualizations of true anomalies caught using the historical average and DBSCAN method applied to Ubon Ratchathani). Of the 9 methods, 4 methods were unable to identify the labelled outbreaks. These methods are density-based profiling with DBSCAN, density-based profiling with DBSCAN including temperature and precipitation data, unsupervised clustering with tsclust [37], and monthly case comparison. The method reporting the most alerts at 32630 is the weekly 3 year median while the method reporting the lowest number of alerts at 5 is density-based profiling with DBSCAN.

**Table 3:** Results from method comparison

Method	Ubon (2014)^3^	Ubon (2015)^3^	Yala (2016)	SSK (2017)^3^	KCN (2017)^3^	KCN (2022)	Tak (2022)	Verified Anomalies detected (%)	Total Reported^4^
Statistical profiling	✘^5^	✘	✔^6^	✘	✘	✘	✘	1 (14%)	882
Predictive confidence interval	✔	✘	✔	✔	✘	✘	✘	3 (43%)	2356
Unsupervised clustering	✘	✘	✘	✘	✘	✘	✘	0 (0%)	75
Density-based profiling	✘	✘	✘	✘	✘	✘	✘	0 (0%)	5
Density-based profiling w/T&P^1^	✘	✘	✘	✘	✘	✘	✘	0 (0%)	452
Historical average	✘	✔	✔	✔	✔	✔	✔	6 (86%)	10875
Weekly Case previous year	✔	✘	✔	✔	✔	✔	✔	6 (86%)	30449
Monthly case 4 years	✘	✘	✘	✘	✘	✘	✘	0 (0%)	5577
Weekly 3 year median^2^	✔	✔	✔	✔	✘	✔	✔	6 (86%)	32630

^1^Temperature and precipitation included in analysis

^2^Baseline method used in Thailand (BIOPHICS)

^3^Ubon: Ubon Ratchathani, SSK: SI Sa Ket, KCN: Kanchanaburi

^4^Total anomalies repored for each method when applied to all malaria cases between 2012 and 2022

^5^Symbol showing that this method was not able to trigger anomaly alerts at least 14 days before this verified outbreak observation

^6^Symbol showing that this method was able to trigger anomaly alerts at least 14 days before this verified outbreak observation

### Code structure and functionalization

After the algorithms’ performance were tested, they were converted into functions with easily adaptable outbreak definitions. Data handling and filtering functions are created to allow user input into the analysis. Additional functions were created to run anomaly detection algorithms across all provinces based on user-defined inputs, such as malaria species and time period for analysis, and to store the anomaly status of each province for map visualization. The code structure (see Fig. 2) was achieved. All the code files can also be found here: https://github.com/mghDissertation/malaria_anomaly_detect.

### User interface

**Fig. 3:**
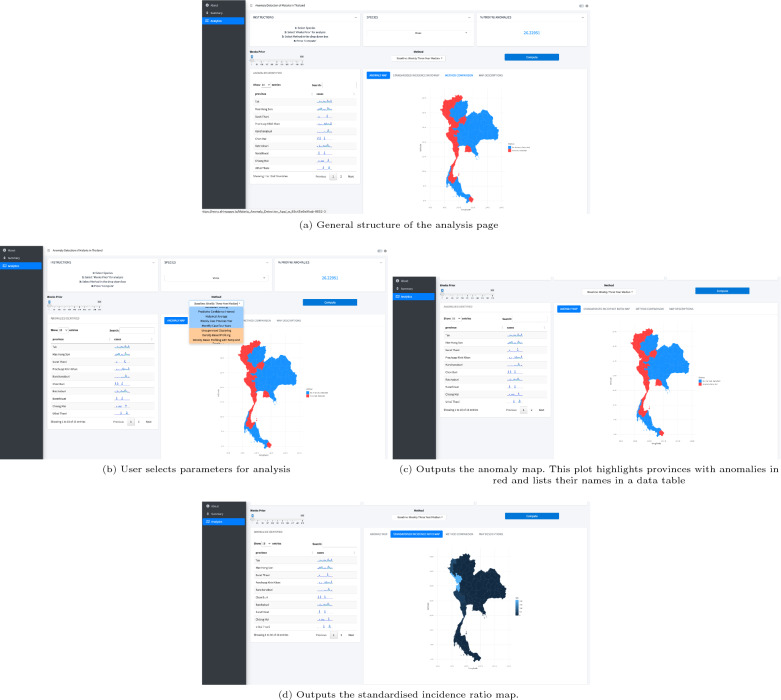
Final user interface. Output from the “Analytics” tab showing maps, anomalous province names, and case trends in the application https://moru.shinyapps.io/Malaria_Anomaly_Detection_App/. The user selects the species, method, and time period of interest to run the analysis. The methods are grouped by machine-learning-based (orange) and threshold or statistical-based (blue). After the investigation is complete, the anomaly map, the standardized incidence ratio map, and the anomalous provincial names are shown. Additional map descriptions are shown in the map descriptions tab

To aid in developing the optimal design and layout for the final dashboard, a wireframe was developed (refer to Additional file 5), specifically tailored for anomaly detection. An intermediate application (refer to Additional file 5) was used to validate code functionality, offering a visual depiction of provinces marked for unusual malaria activity. The dashboard’s design was refined based on feedback from BIOPHICS and fellow researchers, ensuring effective anomaly detection and granting users the flexibility to choose essential parameters. The final dashboard contains three main pages with information on methods, generated visuals, and method-specific accuracy. The aim is to allow users to easily compare different methods, species, and time frames used for analysis. The final dashboard, as shown in Fig. 3 and Additional file 5, will feature the best method on its summary page for DVBD’s use. The final application is hosted here: https://moru.shinyapps.io/Malaria_Anomaly_Detection_App/.

## Discussion

The creation of effective anomaly detection algorithms combined with a user inter-face tailored for anomaly detection supports progress towards the Thailand Malaria Elimination Programme.

### Algorithms

Through the evaluation of algorithms, it was found that three methods—historical average, weekly case comparison, and weekly 3 year median—successfully identified 86% of the labeled outbreaks. However, these methods varied significantly in their alert efficiency, with the total number of alerts generated to verified anomalies detected being 1813, 5075, and 5438, respectively. As observed in Table 3, these three methods detected 6 out of 7 outbreaks. The historical average method was able to detect all verified outbreak dates except for the 2014 Ubon Rachathani outbreak. Given that the dataset begins in 2012 and the historical average method requires data from the previous 3 years, the alert threshold value might have been set higher than intended, preventing the alert from being triggered. In contrast, the weekly case comparison method identified all verified outbreaks except for the 2015 Ubon Ratchathani observation. The weekly case comparison method relies on the weekly cumulative counts from the previous year and because an outbreak was reported in Ubon Ratchathani from the previous year, a slight decrease in case values would not have been able to trigger an alert for this method even if an outbreak was declared. Similarly, the 3 year median method identified all outbreaks except for the one in Kanchanaburi in 2017. Since this outbreak was reported at a subdistrict level, it was more difficult to catch these irregularities when the analysis was completed at a provincial level. Other methods that failed to detect this subdistrict outbreak include statistical profiling, predictive confidence interval, unsupervised clustering with tsclust package [37], density-based profiling using only case data, density-based profiling combining case data with temperature and precipitation data, and monthly case comparison. Although the currently implemented 3 year median method identified 6 out of 7 actual outbreaks, it generated approximately three times as many predictions (or total reported alerts) compared to the historical average method. The primary objective of these algorithms is to guide the DVBD on which areas to prioritize, especially in resource-limited scenarios, to pre-emptively control potential outbreaks. In practice, a low false positive rate combined with a high true positive rate is crucial for DVBD to effectively respond to outbreaks.

The statistical profiling method detected 14.2% of the labelled outbreaks, while the predictive confidence interval method detected 43%. Despite having fewer alerts, the statistical profiling and predictive confidence interval methods reported a ratio of the total number of alerts generated to verified anomalies detected of 882 and 785. For instance, the statistical profiling method identified anomalies solely for the 2016 Yala outbreak. In contrast, the predictive confidence interval method detected the 2016 Yala outbreak and also the 2017 Si Sa Ket and 2014 Ubon Ratchathani outbreaks. By collaborating further with the DVBD, acceptable false positive rates and sensitivity levels can be determined. This will help in refining the customization of warning methods for specific health districts. In the context of clustering-based methods, this analysis found that techniques such as clustering with tsclust [37] and DBSCAN using malaria case data were ineffective in identifying any labelled outbreak data. This was also the case when combining malaria case data with precipitation and temperature metrics. While these methods were tested at a provincial level, their outcomes might vary when implemented at district or village levels.

Compared to time series-based methods, clustering-based anomaly detection methods showed lower accuracy in identifying verified outbreaks when tested with malaria data from 2012 to 2022. Table 3 shows how different methods were able to capture different anomalous activities.

Through further visualization of these methods applied to the Kanchanaburi province (Additional file 14) it becomes evident why some methods are able to capture more anomalies than others. The historical average and statistical profiling use daily malaria cases for analysis and can capture seasonal changes in malaria cases more than the predictive confidence interval method. The statistical profiling method was only able to capture one verified anomaly since the threshold use to classify anomalous observations was much higher than other methods using daily malaria cases. As a result, less anomalies are reported, and fewer verified outbreaks are caught. Compared to the statistical profiling method and the predictive confidence interval method, the historical average method has a lower threshold that outlines the general shape of the daily cases and as a result, more anomalies are reported, and more verified outbreaks are caught early. This is similarly observed in the weekly case comparison and weekly 3 year median methods. These two methods use weekly malaria cases and can capture malaria seasonality while creating a threshold which is high enough that not all observations are anomalous, but low enough to capture weeks with higher than usual malaria cases. As a result, these two methods had a better performance than other methods. Similar to the statistical profiling method, the monthly case comparison method is capable of identifying seasonal malaria trends. However, its threshold is significantly higher than the observed data, attributable to the elevated averages of malaria cases from the preceding 4 years. As a result, the monthly case comparison method captured few anomalies and was not able to capture any of the verified outbreaks. Figure 2 shows that clustering-based methods defined observations at large malaria peaks as anomalous. These methods were not able to capture anomalies between peaks and for smaller malaria waves that preceded larger ones.

A combination of these methods can be used to capture different types of anomalies across countries with a similar malaria profile to Thailand and should be tested with more verified outbreak dates. In this context, the historical average method outperformed others due to its high accuracy in identifying outbreaks and its low false positive rate. Observations deemed anomalous are categorized based on threshold definitions. These thresholds can be adjusted to match the tolerance levels set by health districts, comparable to the criteria used for SMC area identification. Depending on the application and scenario, tailored algorithm thresholds can be designed based on health district needs. Easy integration is possible as all methods and code are functionalized and adaptable to requirements set by different health districts.

### User interface

The final dashboard, tailored specifically for anomaly detection, has been designed to be user-friendly, allowing disease surveillance professionals to easily navigate and interact with the detection algorithms. It offers tools for visualizing anomalies and user-defined analysis parameters, and it facilitates in-depth analysis of atypical patterns in malaria data.

The dashboard application has three main pages. The ‘Introduction’ page presents the application’s objectives and methodologies. The ‘Summary’ page provides weekly insights on anomalous provinces and malaria cases, categorized by border types, based on a default method determined by the health district.

The analysis page allows users to expand their analysis through user-defined methods, malaria species, and time frames. Its core aim is to showcase how different methods and time frames affect provincial alerts. Method options are grouped into clustering-based (orange) or time series-based (blue) in a dropdown methods section in the analysis page of the dashboard.

The analysis page provides step-by-step guidance, highlighting anomalous provinces on a map and showing standardized malaria incidence across Thailand. After each analysis, anomalous provinces are listed, and an interactive widget displays malaria cases over time per province.

## Limitations

Certain limitations were present in this study. Specific statistical methods relied on literature to classify anomalies as values surpassing 3 standard deviations above the mean. As each province follows its own protocol for defining malaria out-breaks and resource allocation, collaborating with different health districts to establish outbreak thresholds is essential to identify the most suitable method for them. This cooperative approach, combined with user feedback for both the algorithms and user interface, can help identify the most suitable anomaly detection method for each province. For the dataset used, observations started in 2012 and ended in May 2022, and lacks real-time integration with the malaria reporting database. Although functions are compatible with raw data, real-time integration should be conducted. While this analysis focused developing a proof-of-concept on a provincial level for efficiency, it could be extended to subdistrict or subvillage scales to represent the surveillance resolution implemented in the 1-3-7 program. More outbreak data points and working directly with the DVBD surveillance team would improve validation, algorithm sensitivity, and the final interface.

## Conclusions

An enhanced early warning system is proposed to bolster malaria elimination efforts in regions where malaria is endemic, such as certain areas of Thailand. Clustering-based and time series-based methods were developed and compared. Compared to the current method analysing malaria case data from 2012 to 2022, the historical average-based method demonstrated equivalent sensitivity with a reduced false positive rate. A user interface tailored for anomaly detection is developed and aids in early detection by summarizing anomalies on a weekly basis across provinces. The code has been optimized for functionality and is configured to synchronize with the real-time malaria database. The anomaly detection algorithms could be integrated at the case identification stage of the 1-3-7 protocol and applied at a sub village level. This approach would assist in determining the allocation of resources to prevent the spread of atypical malaria cases. The proposed early warning system enhances the timely identification of provinces at risk of epidemics and seamlessly integrates with Thailand’s malaria surveillance system.

### Supplementary Information

**Additional file 1: ** List of Provinces in Thailand.

**Additional file 2: **Summary of Malaria Case, Temperature, and Precipitation Data.

**Additional file 3: **Monthly Case Comparison Methods.

**Additional file 4: **Algorithm Validation Example with the Ubon Ratchathani Province.

**Additional file 5: **Interface Development: Wireframe, Intermediate Application, and Final Application.

**Additional file 6: **List of the confirmed outbreaks.

**Additional file 7: **The precipitation data.

**Additional file 8: **The temperature data.

**Additional file 9: **The provincial population data.

**Additional file 10: **A High-Level Overview of the Code Structure.

**Additional file 11: **Details of Anomaly Detection Methods Used for Analysis.

**Additional file 12: **Detailed Descriptions of Verified Outbreaks.

**Additional file 13: **The pseudocode for validating and comparing various anomaly detection methods.

**Additional file 14: **Visualisation of Thresholds for Time Series Methods.

## Data Availability

The Thai malaria data is not publishable, however, a summary of the data is found in Additional file 2. Additional file 6 outlines the outbreak dates used for the method comparison section. The daily precipitation data can be downloaded from the Temperature and Precipitation Gridded Data for Global and Regional Domains Derived from In-situ and Satellite Observations from the Copernicus Climate Data Store (https://cds.climate.copernicus.eu/cdsapp#!/dataset/insitu-gridded-observations-global-and-regional?tab=overview) and the daily temperature data can be downloaded from Berkeley Earth’s Global Temperature Gridded Data (https://berkeleyearth.org/data/). The precipitation and temperature data is visualized in Additional file 2. Additional file 7 and 8 shows the precipitation and temperature data used for this report. The provincial population data is available through the National Statistical Office (http://statbbi.nso.go.th/staticreport/page/sector/en/01.aspx) (details in Additional file 9).

## Abbreviations

DVBD: Division of Vector Borne Disease
eMIS: Electronic Malaria Information System
BIOPHICS: Center of Excellence for Biomedical and Public Health Informatics
DBSCAN: Density-Based Spatial Clustering of Applications with Noise
WHO: World Health Organization
SMC: Seasonal Malaria Chemoprevention
ARIMA: Autoregressive Integrated Moving Average

## Code availability

The code is available on Github at (https://github.com/mghDissertation/malaria_anomaly_detect).

## References

[1] WHO (2023). Fact sheet about malaria.

[2] Chareonviriyaphap T, Bangs MS, Ratanatham S (2000). Status of malaria in Thailand. Southeast Asian J Trop Med Public Health.

[3] WHO (2023). Thailand gears up to eliminate malaria by 2024.

[4] Lertpiriyasuwat C, Sudathip P, Kitchakarn S, Areechokchai D, Naowarat S, Shah JA (2021). Implementation and success factors from Thailand’s 1-3-7 surveillance strategy for malaria elimination. Malar J.

[5] Ma S, Lawpoolsri S, Soonthornworasiri N, Khamsiriwatchara A, Jandee K, Taweeseneepitch K (2016). Effectiveness of implementation of electronic malaria information system as the national malaria surveillance system in Thailand. JMIR Public Health Surveill.

[6] Shah JA (2022). Learnings from Thailand in building strong surveillance for malaria elimination. Nat Commun.

[7] Jongdeepaisal M, Khonputsa P, Prasert O, Maneenet S, Pongsoipetch K, Jatapai A (2022). Forest malaria and prospects for anti-malarial chemoprophylaxis among forest goers: findings from a qualitative study in Thailand. Malar J.

[8] Smithuis FM, White NJ (2022). Spend wisely to eliminate malaria. Lancet Infect Dis.

[9] Maharaj R (2017). Early warning systems for the detection of malaria outbreaks. Indian J Med Res.

[10] Konchom S, Singhasivanon P, Kaewkungwal J, Chuprapawan S, Thimasarn K, Kidson C (2006). Early detection of malaria in an endemic area: model development. Southeast Asian J Trop Med Public Health.

[11] Thailand Malaria Elimination Programme. https://malaria.ddc.moph.go.th/malariar10/index_newversion.php

[12] Schneider P, Xhafa F (2022). Anomaly detection and complex event processing over IoT data streams.

[13] Pang J, Liu D, Peng Y, Peng X (2018). Optimize the coverage probability of prediction interval for anomaly detection of sensor-based monitoring series. Sensors.

[14] Salik JFN. Sub-band anomaly detection and spatial localization. In: 2007 IEEE Northeast Workshop on Circuits and Systems. 2007

[15] Syarif I, Prugel-Bennett A, Wills G, Benlamri R (2012). Unsupervised clustering approach for network anomaly detection. Networked digital technologies.

[16] Thang TM, Kim J. The anomaly detection by using DBSCAN clustering with multiple parameters. In: International Conference on Information Science and Applications. 2011.

[17] Samariya D, Ma J, Aryal S, Zhao X (2023). Detection and explanation of anomalies in healthcare data. Health Inf Sci Syst.

[18] Nanehkaran YA, Licai Z, Chen J, Jamel AAM, Shengnan Z, Navaei YD (2022). Anomaly detection in heart disease using a density-based unsupervised approach. Wireless Commun Mobile Comput.

[19] Röchner P, Rothlauf F (2023). Unsupervised anomaly detection of implausible electronic health records: a real-world evaluation in cancer registries. BMC Med Res Methodol.

[20] Bijlani N, Nilforooshan R, Kouchaki S (2022). An unsupervised data-driven anomaly detection approach for adverse health conditions in people living with dementia: cohort study. JMIR Aging.

[21] Eze PU, Geard N, Mueller I, Chades I (2023). Anomaly detection in endemic disease surveillance data using machine learning techniques. Healthcare.

[22] Thailand - Climatology. Climate Change Knowledge Portal. https://climateknowledgeportal.worldbank.org/country/thailand/climate-data-historical

[23] Kiguchi M, Takata K, Hanasaki N, Archevarahuprok B, Champathong A, Ikoma E (2021). A review of climate-change impact and adaptation studies for the water sector in Thailand. Environ Res Lett.

[24] World Bank Climate Change Knowledge Portal- Thailand. https://climateknowledgeportal.worldbank.org/country/thailand

[25] Dabaro D, Birhanu Z, Negash A, Hawaria D, Yewhalaw D (2021). Effects of rainfall, temperature and topography on malaria incidence in elimination targeted district of Ethiopia. Malar J.

[26] Oheneba-Dornyo TV, Amuzu S, Maccagnan A, Taylor T (2022). Estimating the impact of temperature and rainfall on malaria incidence in Ghana from 2012 to 2017. Environ Model Assess.

[27] Tiu LA, Wahid WE, Andriani WY, Mirnawati M, Tosepu R (2021). Literature review: impact of temperature and rainfall on incident malaria. IOPSci Conf Ser.

[28] Copernicus Climate Change Service (2021). Temperature and precipitation gridded data for global and regional domains derived from in-situ and satellite observations. ECMWF.

[29] Berkeley Earth. Environmental science, data, and analysis of the highest qualityIndependent, non-governmental, and open-source. https://berkeleyearth.org/

[30] Ammatawiyanon L, Tongkumchum P, Lim A, McNeil D (2022). Modelling malaria in southernmost provinces of Thailand: a two-step process for analysis of highly right-skewed data with a large proportion of zeros. Malar J.

[31] Canva. Canva. https://www.canva.com/

[32] Chang W. Shiny. https://www.rdocumentation.org/packages/shiny/versions/1.7.4.1

[33] raster. https://cran.r-project.org/web/packages/raster/index.html

[34] South A. rworldmap. https://github.com/AndySouth/rworldmap/

[35] ggplot2. https://cran.r-project.org/web/packages/ggplot2/index.html

[36] Granjon D. bs4Dash. https://rinterface.github.io/bs4Dash/index.html

[37] Montero P, Vilar J (2014). TSclust: an R package for time series clustering. J Stat Softw.

[38] Mullineaux DR, Irwin G (2017). Error and anomaly detection for intra-participant time-series data. Int Biomech.

[39] Nekorchuk DM, Gebrehiwot T, Lake M, Awoke W, Mihretie A, Wimberly MC (2021). Comparing malaria early detection methods in a declining transmission setting in northwestern Ethiopia. BMC Public Health.

[40] Hay SI, Simba M, Busolo M, Noor AM, Guyatt HL, Ochola SA (2002). Defining and detecting malaria epidemics in the highlands of Western Kenya. Emerg Infect Dis.

[41] Cullen JR, Chitprarop U, Doberstyn EB, Sombatwattanangkul K (1984). An epidemiological early warning system for malaria control in northern Thailand. Bull World Health Organ.

[42] Kulanuwat L, Chantrapornchai C, Maleewong M, Wongchaisuwat P, Wimala S, Sarinnapakorn K (2021). Anomaly detection using a sliding window technique and data imputation with machine learning for hydrological time series. Water.

[43] Kozitsin V, Katser I, Lakontsev D (2021). Online forecasting and anomaly detection based on the ARIMA model. Appl Sci.

[44] Ye F, Liu Z, Liu Q, Wang Z (2020). Hydrologic time series anomaly detection based on Flink. Math Probl Eng.

[45] Xue S, Chen H, Zheng X (2022). Detection and quantification of anomalies in communication networks based on LSTM-ARIMA combined model. Int J Mach Learn Cyber.

[46] Sahu RT, Verma MK, Ahmad I (2023). Density-based spatial clustering of application with noise approach for regionalisation and its effect on hierarchical clustering. Int J Hydrol Sci Technol.

[47] Hahsler M. dbscan: density-based spatial clustering of applications with noise (DBSCAN) and related algorithms. https://cran.r-project.org/web/packages/dbscan/index.html

[48] Roh M, Lausatianragit K, Chaitaveep N, Jongsakul K, Sudathip P, Raseebut C (2021). Civilian-military malaria outbreak response in Thailand: an example of multi-stakeholder engagement for malaria elimination. Malar J.

[49] WHO. Programmatic review of the national malaria programme in Thailand: summary report. WHO Regional Office for South-East Asia; 2016. https://apps.who.int/iris/handle/10665/253958

[50] Guide to Malaria Elimination For Thailand’s Local Administrative Organizations and the Health Network. Bureau of Vector Borne Diseases, Department of Disease Control, Ministry of Public Health

[51] Mercado CEG, Lawpoolsri S, Sudathip P, Kaewkungwal J, Khamsiriwatchara A, Pan-ngum W (2019). Spatiotemporal epidemiology, environmental correlates, and demography of malaria in Tak Province, Thailand (2012–2015). Malar J.

