# Supplementary material for: Early warning systems for malaria outbreaks in Thailand: an anomaly detection approach
Source: Malar J. 2024 Jan 8;23:11. doi: 10.1186/s12936-024-04837-x (PMC10775623; doi:10.1186/s12936-024-04837-x)
Supplement: Supplementary file 1 — Additional file 1: List of Provinces in Thailand. [file 12936_2024_4837_MOESM1_ESM.pdf]

Provinces and their Corresponding ID

Province Name	Province ID
Bangkok Metropolis	10
Samut Prakan	11
Nonthaburi	12
Pathum Thani	13
Phra Nakhon Si Ayutthaya	14
Ang Thong	15
Lop Buri	16
Sing Buri	17
Chai Nat	18
Saraburi	19
Chon Buri	20
Rayong	21
Chanthaburi	22
Trat	23
Chachoengsao	24
Prachin Buri	25
Nakhon Nayok	26
Sa Kaeo	27
Nakhon Ratchasima	30
Buri Ram	31
Surin	32
Si Sa Ket	33
Ubon Ratchathani	34
Yasothon	35
Chaiyaphum	36
Amnat Charoen	37
Bueng Kan	38
Nong Bua Lam Phu	39
Khon Kaen	40
Udon Thani	41
Loei	42
Nong Khai	43
Maha Sarakham	44
Roi Et	45
Kalasin	46
Sakon Nakhon	47
Nakhon Phanom	48
Mukdahan	49
Chiang Mai	50
Lamphun	51
Lampang	52

Uttaradit	53
Phrae	54
Nan	55
Phayao	56
Chiang Rai	57
Mae Hong Son	58
Nakhon Sawan	60
Uthai Thani	61
Kamphaeng Phet	62
Tak	63
Sukhothai	64
Phitsanulok	65
Phichit	66
Phetchabun	67
Ratchaburi	70
Kanchanaburi	71
Suphan Buri	72
Nakhon Pathom	73
Samut Sakhon	74
Samut Songkhram	75
Phetchaburi	76
Prachuap Khiri Khan	77
Nakhon Si Thammarat	80
Krabi	81
Phangnga	82
Phuket	83
Surat Thani	84
Ranong	85
Chumphon	86
Songkhla	90
Satun	91
Trang	92
Phatthalung	93
Pattani	94
Yala	95
Narathiwat	96

Table 1: Provincial English names and their province ID

Table 1 shows the English name of all the provinces in Thailand and their provincial ID.

Figure 1 shows all the Thai provinces and their corresponding provincial IDs.

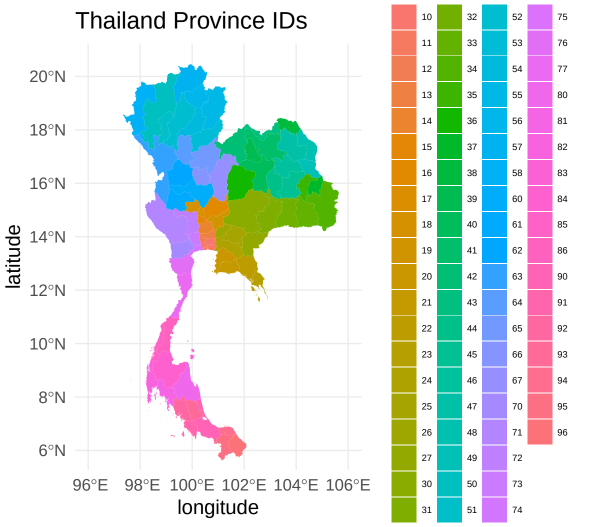

Fig. 1: Provinces in Thailand and their provincial ID
