# Supplementary material for: Early warning systems for malaria outbreaks in Thailand: an anomaly detection approach
Source: Malar J. 2024 Jan 8;23:11. doi: 10.1186/s12936-024-04837-x (PMC10775623; doi:10.1186/s12936-024-04837-x)
Supplement: Supplementary file 2 — Additional file 2: Summary of Malaria Case, Temperature, and Precipitation Data. [file 12936_2024_4837_MOESM2_ESM.pdf]

Summary of Malaria Case, Temperature, and Precipitation Data

Province IDs	Total Malaria Cases (2012 - 2022)
17 , 18 , 43 , 15 , 38 , 26 , 39 , 56 , 16 , 66 , 54 , 42 , 53 , 75 , 14 , 64 , 19	1 - 30
36 , 44 , 12 , 48 , 55 , 45 , 60 , 13 , 40 , 37, 11, 41, 62, 92, 93, 74 , 72, 51, 52, 67 , 73	31 - 100
31 , 83, 65, 61, 91 , 35 , 81 , 49, 80, 24, 30, 47, 20, 10, 21, 46 , 27, 25, 94	101 - 1000
23 , 32 , 57 , 82 , 50, 22	1001 - 2000
86 , 76, 96 , 70 , 77, 85 , 84, 90	2001 - 7000
33, 58	7001 - 10000
71 , 34 , 95, 63	10001 - 62000

Table 1: Total Cumulative Malaria Cases by Provincial ID

Data used for this project is provided from BIOPHICS. Table 1 shows the total malaria cases by provincial ID.

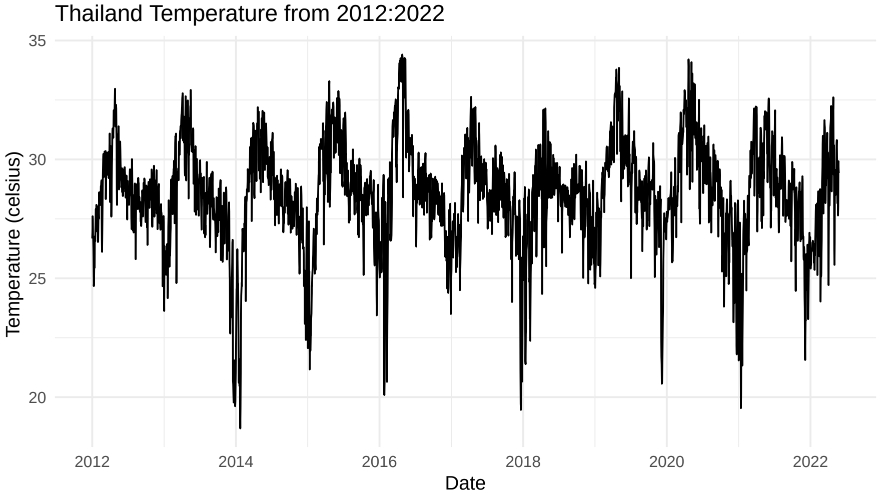

Fig. 1: Temperature data used for analysis from 2012 to 2021

Daily Thai temperature data is extracted from Berkeley Earth's Global Temperature Gridded Data and is combined with malaria case data for one unsupervised clustering anomaly detection method. Figure 1 shows the temperature data used.

Daily Thai precipitation data from the Copernicus Climate Data Store is combined with malaria case data for one unsupervised clustering anomaly detection method. Figure 2 shows the precipitation data used.

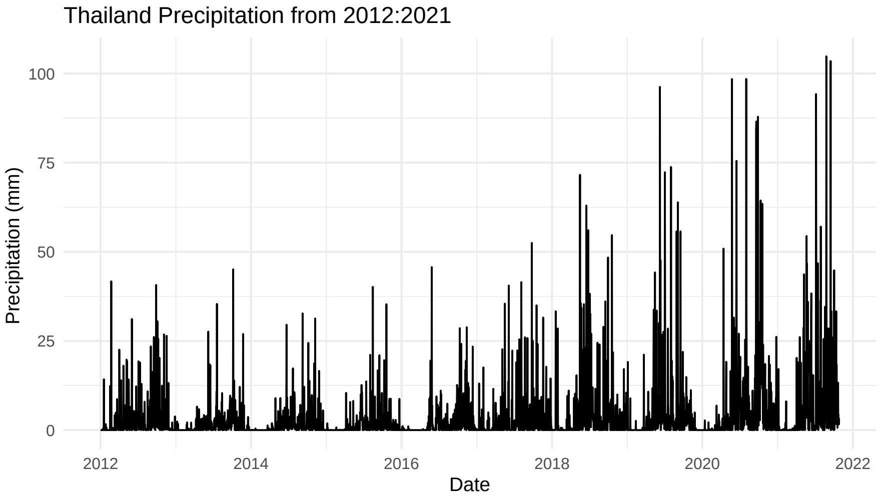

Fig. 2: Precipitation data used for analysis from 2012 to 2022
