# Supplementary material for: Early warning systems for malaria outbreaks in Thailand: an anomaly detection approach
Source: Malar J. 2024 Jan 8;23:11. doi: 10.1186/s12936-024-04837-x (PMC10775623; doi:10.1186/s12936-024-04837-x)
Supplement: Supplementary file 3 — Additional file 3: Monthly Case Comparison Methods. [file 12936_2024_4837_MOESM3_ESM.pdf]

# Monthly Case Comparison Method

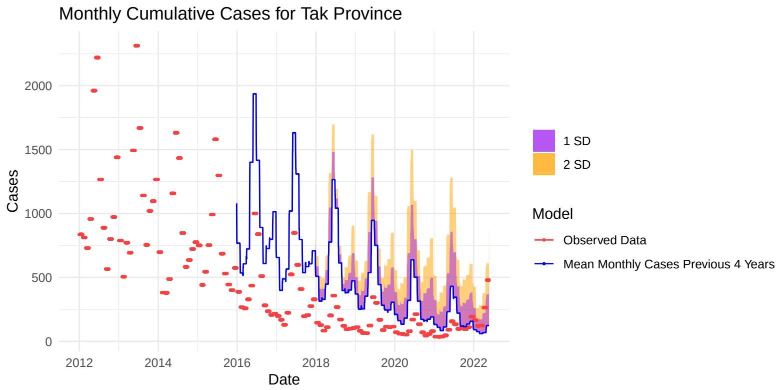


**Fig. 1**: Implementing the monthly case comparison method for the Tak province from 2012 to 2022

Figure 1 shows the monthly case comparison method for the Tak province. Observations from 2012 to 2016 do not have mean monthly cumulative case values since data from the previous 4 years is required for this method and the dataset used is only has data available from 2012 onward. The blue trend line shows the monthly cumulative cases averaged over the previous 4 years while the purple and orange shaded regions show 1 and 2 standard deviations bands above the mean, respectively. Observations (red) above 2 standard deviations (orange) are classified as anomalous.

# Historical Average Method

Figure 2 shows the historical average method implemented on the Tak province. The mean value trend line from 2012 to 2015 is not available since the mean value is calculated from the previous 3 years and the data is only available from 2012. The rolling mean value from the previous 3 years is shown as the blue trend line while the current daily observations are shown as red points. Observations (red) falling above the blue trend line is classified as anomalous.

# Three-Year Median Baseline Method

Figure 3 shows the weekly median value method applied to the Tak province. The weekly median value from the previous three years is shown as the blue trend line and starts in 2013 since a minimum of the previous year data is required. If weekly cumulative observations (red) are above the blue trend line, that point is classified as anomalous.

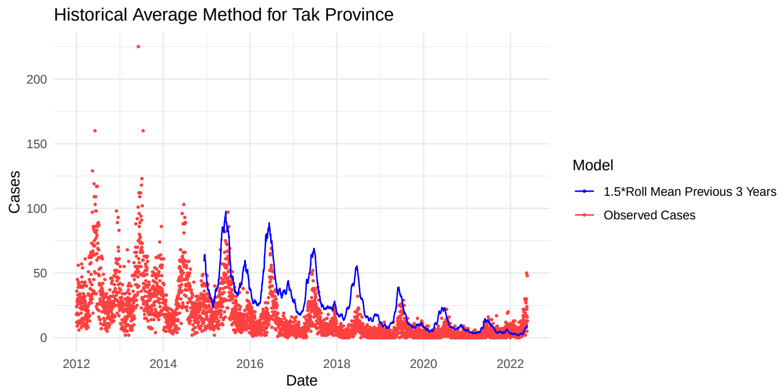


**Fig. 2**: Implementing the historical average method for the Tak province from 2012 to 2022

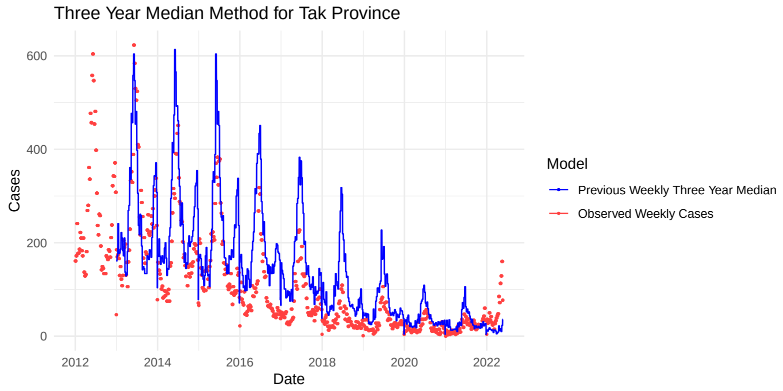


**Fig. 3**: Implementing the weekly three-year median method for the Tak province from 2012 to 2022
