# Supplementary material for: Early warning systems for malaria outbreaks in Thailand: an anomaly detection approach
Source: Malar J. 2024 Jan 8;23:11. doi: 10.1186/s12936-024-04837-x (PMC10775623; doi:10.1186/s12936-024-04837-x)
Supplement: Supplementary file 4 — Additional file 4: Algorithm Validation Example with the Ubon Ratchathani Province. [file 12936_2024_4837_MOESM4_ESM.pdf]

Algorithm Validation Example with the Ubon Ratchathani Province

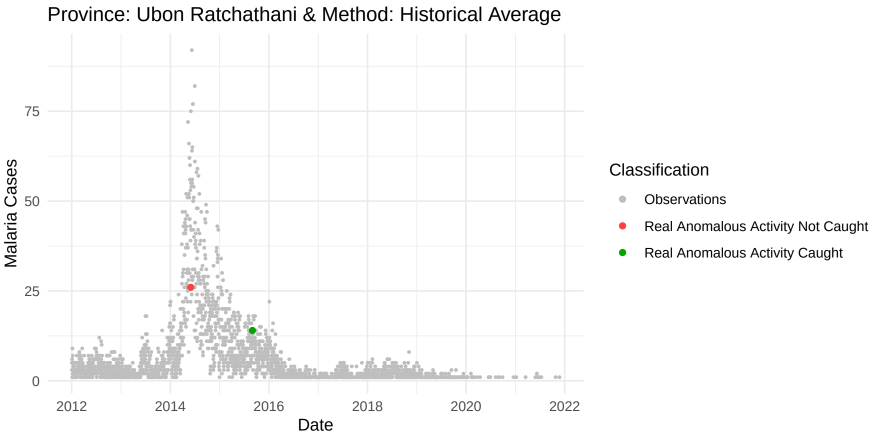

Fig. 1: Visualisation of the true anomalies caught using the historical average method for Ubon Ratchathani from 2012 - 2022

Figure 1 shows true anomalies that were caught using the historical average method for Ubon Ratchathani. Using outbreak dates based on literature in the algorithm validation and comparison section, outbreak dates caught by this method are shown in green and ones that were not caught by this method are shown in red.

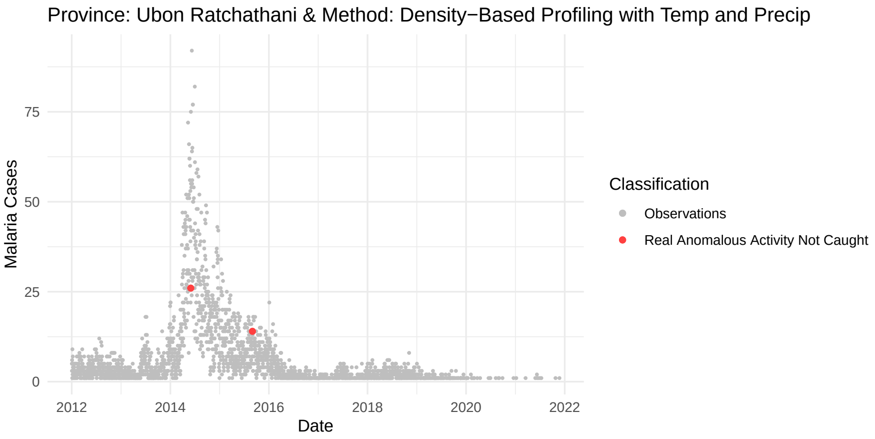

Fig. 2: Visualisation of the true anomalies caught using Density-Based Profiling with temperature and precipitation data for Ubon Ratchathani from 2012 to 2022

Figure 2 shows the Density-Based Profiling method with malaria, temperature, and precipitation data applied to Ubon Ratchathani. This figure shows two of the true anomalies were not caught by this method for this province as they are red.
