# Supplementary material for: Early warning systems for malaria outbreaks in Thailand: an anomaly detection approach
Source: Malar J. 2024 Jan 8;23:11. doi: 10.1186/s12936-024-04837-x (PMC10775623; doi:10.1186/s12936-024-04837-x)
Supplement: Supplementary file 5 — Additional file 5: Interface Development: Wireframe, Intermediate Application, and Final Application. [file 12936_2024_4837_MOESM5_ESM.pdf]

Interface Development: Wireframe, Intermediate Application, and Final Application

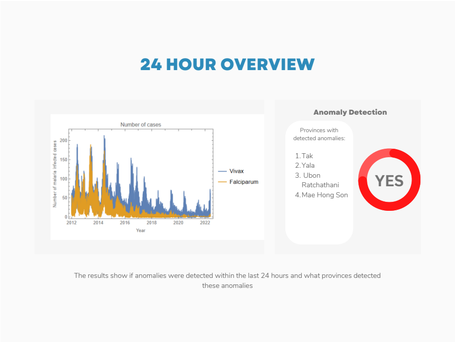

(a) Main page

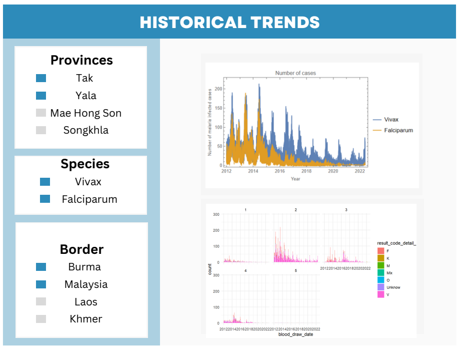

(b) Analysis page

Fig. 1: Wireframe of the app created in Canva

Figure 1 shows the wireframe of the app made in Canva. Figure 2 shows the intermediate R Shiny application used for testing all functions together and Figure 3 - 4 shows the final dashboard.

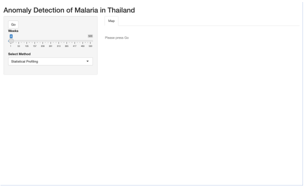

(a) Initial interface before pressing "Go"

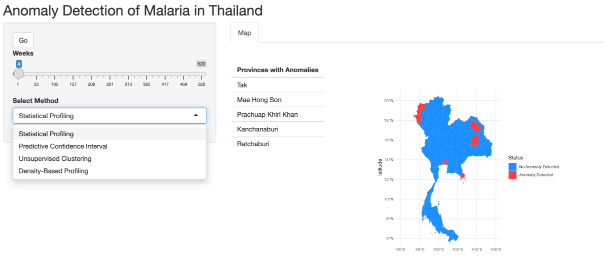

(b) Final interface after pressing "Go". Highlights anomalous provinces

Fig. 2: Intermediate app made with R Shiny to test functions

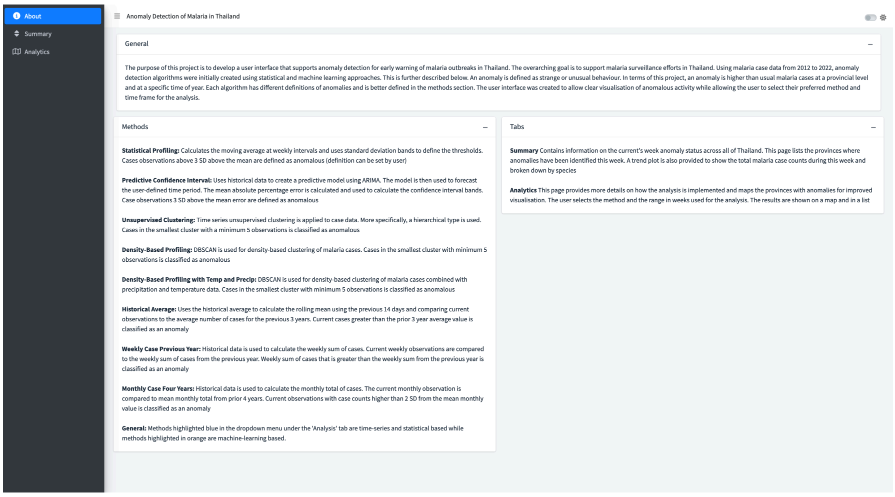

Fig. 3: The first page of the final dashboard. This is the “About” tab and contains general information about the project and the methods used in the “Analysis” tab.

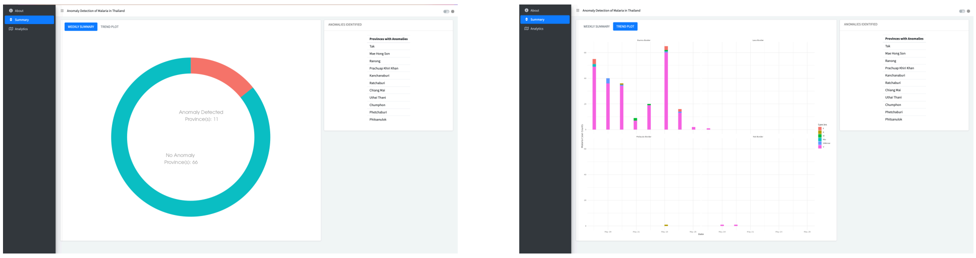

(a) Initial graphic showing ratio of provinces with and without anomalies

(b) Border and species trend plots for the weekly summary

Fig. 4: This is the second page under the “Summary” Tab. This page provides a weekly overview of anomalous activity captured in the current week. The method used for this analysis is the historical average method
