# Supplementary material for: Early warning systems for malaria outbreaks in Thailand: an anomaly detection approach
Source: Malar J. 2024 Jan 8;23:11. doi: 10.1186/s12936-024-04837-x (PMC10775623; doi:10.1186/s12936-024-04837-x)
Supplement: Supplementary file 10 — Additional file 10: A High-Level Overview of the Code Structure. [file 12936_2024_4837_MOESM10_ESM.pdf]

A High-Level Overview of the Code Structure

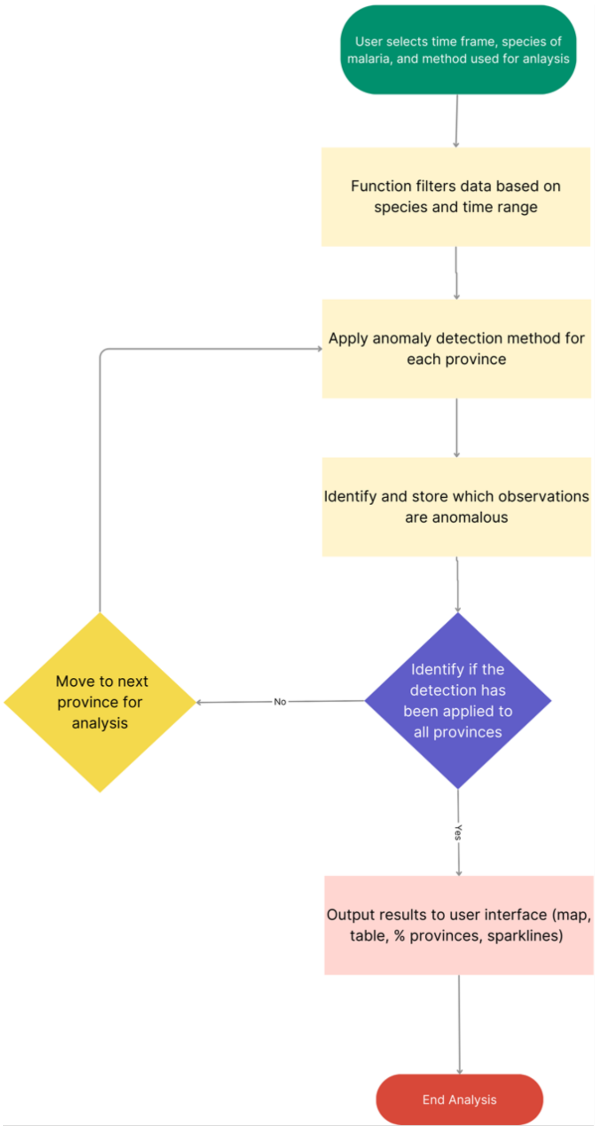

Fig. 1: High-level flow diagram of the code structure and main functions for the back-end of the code

Figure 1 shows a high-level flow diagram of the code structure and main functions focusing on the back-end of the code and how it interacts with the interface.
