# Supplementary material for: Early warning systems for malaria outbreaks in Thailand: an anomaly detection approach
Source: Malar J. 2024 Jan 8;23:11. doi: 10.1186/s12936-024-04837-x (PMC10775623; doi:10.1186/s12936-024-04837-x)
Supplement: Supplementary file 11 — Additional file 11: Details of Anomaly Detection Methods Used for Analysis. [file 12936_2024_4837_MOESM11_ESM.pdf]

Details of Anomaly Detection Methods Used for Analysis

1 Statistical Profiling

Statistical profiling uses the moving average of previous malaria data to create statistical standard deviation bands that define upper thresholds [1, 2]. This method uses daily aggregated malaria case data for analysis. Data points falling outside the upper threshold of 3 standard deviations above the mean are classified as anomalous. Variations in standard deviation thresholds have been used previously to define outbreaks [3, 4, 5].

$$x_i = \text{rollmean}(z, k)_i = \frac{1}{k} \sum_{j=i-\lfloor \frac{k-1}{2} \rfloor}^{i+\lfloor \frac{k}{2} \rfloor} z_j \tag{1}$$

In Equation 1, z is the vector of malaria cases, k is the length of the sliding window, and i is the index of the current element.

$$\sigma = \sqrt{\frac{1}{n}\sum_{i=1}^{n}(x_i - \bar{x})^2} \tag{2}$$

The standard deviation of the moving average is used to calculate bands for further classification of the observations. In Equation 2, n is the total number of cases for the moving average, x_i is each value of the moving average, and $\bar{x}$ is the mean of the moving average.

$$classification = \begin{cases} Anomalous, & \text{if } z_i \geq x_i + 3\sigma \\ NotAnomalous, & \text{otherwise} \end{cases} \tag{3}$$

2 Predictive Confidence Interval

The predictive confidence interval approach uses a predictive model to create a baseline scenario. This model is used to create thresholds based on the mean absolute percentage error when compared to actual malaria cases for a defined period. The predictive model was built using historical case data using a seasonal autoregressive integrated moving average model or ARIMA model [6]. Seasonal ARIMA models are commonly used for forecasting in R and was selected to capture seasonality of malaria data [7]. A user-defined period is selected for the model to forecast and the absolute error between the predicted and actual values are calculated. Statistical bands are created from the mean error. This method uses daily aggregated malaria case data for analysis. Data points falling outside the upper threshold of 3 standard deviations above the predicted value are classified as anomalous. Algorithm 1 outlines this method.

Algorithm 1 Predictive Confidence Interval

1: Divide the total dataset for the current province into a train set (before the specified date) and a test set (after the specified date)
2: Using ARIMA, create a predictive model using the training data
3: Forecast values using the ARIMA model for the test dataset date range
4: Calculate the error by taking the difference between the actual data and the predictive data points
5: Calculate the mean predictive error using the calculation in Equation 5
6: Calculate the standard deviations of the mean predictive error
7: Classify the observations as anomalous or non-anomalous, similar to Equation 3.
8: Anomalous data points are defined as being 3 standard deviations greater than the predicted value

Equation 4 calculates the error by taking the difference between the model's prediction and the actual observation. The moving average of the error is calculated using Equation 5 and is used to calculate the standard deviation bands for classification, similar to Equation 3.

$$\text{error} = A_t - F_t \tag{4}$$

$$\text{RollError}_i = \text{rollmean}(error, k)_i = \frac{1}{k} \sum_{j=i-\lfloor \frac{k-1}{2} \rfloor}^{i+\lfloor \frac{k}{2} \rfloor} error_j \tag{5}$$

3 Unsupervised Clustering

To implement time series unsupervised clustering, the tsclust() function in the R TSclust library is used [8]. This function measures the dissimilarity between objects in a time series to group similar objects in the same cluster [9]. Through time series clustering, the observations are grouped into anomalous and non-anomalous objects. Another type of unsupervised clustering technique used for outlier detection is Density-Based Spatial Clustering of Applications with Noise algorithm (DBSCAN) [10, 11]. Similar to tsclust, density-based clustering groups observations based on region densities and considers clusters having few data points as anomalous clusters [10]. Two algorithms using DBSCAN are created: one using only daily aggregated malaria case data and another using daily malaria case data and processed and scaled temperature and precipitation data. Once clustered, the algorithm labels data points in the smallest cluster as anomalous.

4 Weekly Case Comparison

The weekly case comparison method is similarly used in a web-based information system known as Malaria Online [12]. This method uses weekly aggregated malaria case data. The weekly incidence is calculated across previous years, and if the current

Algorithm 2 Unsupervised Clustering with DBSCAN

Calculate malaria cases per day
Combine the temperature data, precipitation data, and the malaria case data based on date
Extract the case, temperature, and precipitation data from the dataset
Identify if there more than one observations for cases, temperature, and precipitation
if any of the case, temperature, or precipitation data has a length of 1 **then**
    Conduct clustering using dbscan
else
    Scale the case, temperature, and precipitation data so they are comparable
    Conduct clustering using dbscan
end if
Extract the cluster information
Check if there are more than one cluster so anomalies can be defined
if Only one cluster exists **then**
    No Anomalous Activity is detected
else
    Extract the clusters
    Sort the clusters from smallest to largest
    Classify the anomalous data points as the observations in the smallest cluster
    Match the real data to the cluster it belongs with 1 being classified as anomalous and 0 as non anomalous observations
end if

incidence is higher than the weekly incidence from the previous year, an alert is triggered and classified as anomalous [12]. Observations in 2012 do not have a threshold since data from the previous year is required for comparison and the dataset used is only available for 2012 onward.

In Equation 6, γ is a vector of weekly malaria cases across all available data and i is the index of the current element. If the current weekly value, γ_i, is greater than the weekly value from the previous year, γ_{i-52}, the current element is classified as anomalous.

$$classification = \begin{cases} Anomalous, & \text{if } \gamma_i \geq \gamma_{i-52} \\ NotAnomalous, & \text{otherwise} \end{cases} \tag{6}$$

5 Monthly Case Comparison

Like the weekly incidence comparison, the monthly incidence method calculates the monthly incidence for previous years. This method uses monthly aggregated malaria case data for analysis. If the monthly incidence is higher than 2 standard deviations above the mean values from the previous 4 years, the observation is classified as

anomalous. This method was used as the epidemic threshold in the Sisaket province to report an outbreak in April 2017 [13].

The monthly malaria cases are calculated across all available data and represented as λ. Using the monthly cases, the previous 4-year monthly average, $\lambda_{i,avg4yr}$, is calculated in Equation 7. If the current monthly element is greater than $\lambda_{i,avg4yr}$ plus 2 times the standard deviation of $\lambda_{i,avg4yr}$, represented as $\sigma_{iavg4yr}$, the current element is classified as anomalous. The classification of observations is shown in Equation 8.

$$\lambda_{i,avg4yr} = \text{previous 4 year average}_i = \frac{1}{n}\sum_{j=1}^{n=4} \lambda_{i-12*j} \tag{7}$$

For the classification:

$$classification = \begin{cases} Anomalous, & \text{if } \lambda_i \geq \lambda_{i,avg4yr} + 2\sigma_{iavg4yr} \\ NotAnomalous, & \text{otherwise} \end{cases} \tag{8}$$

6 Rolling Historical Average

The historical average method compares the current observation with the mean observation from the previous 3 years. This method uses daily aggregated malaria case data for analysis. To calculate the mean value from the past three years, the current month and day are used to subset the previous years' data and set as a centre point for analysis. The average number of cases from the previous 7 days and the upcoming 7 days from that centre point is taken and calculated for all three previous years as shown in Equations 9 and 10. The mean for these three values is taken and used as a threshold. If the current case count is greater than the threshold, the observation is classified as anomalous. This classification is shown in Equation 11.

$$\epsilon_i = \text{rollmean}(z,k)_i = \frac{1}{k}\sum_{j=i-\left\lfloor\frac{k-1}{2}\right\rfloor}^{i+\left\lfloor\frac{k}{2}\right\rfloor} z_j \tag{9}$$

$$\epsilon_{i,avg3yr} = \text{previous 3 year average}_i = \frac{1}{n}\sum_{j=1}^{n=3} \epsilon_{i-365*j} \tag{10}$$

$$classification = \begin{cases} Anomalous, & \text{if } \epsilon_i \geq \epsilon_{i,avg3yr} \\ NotAnomalous, & \text{otherwise} \end{cases} \tag{11}$$

7 Weekly Three-Year Median

The weekly three-year median method compares the current weekly incidence to the median value of weekly incidences from the previous three years. This method uses weekly aggregated malaria case data for analysis. This method is currently used in the Thailand Malaria Elimination Program dashboard and will be used as the base method for comparison[14]. Algorithm 3 for this method is shown.

Algorithm 3 Weekly Three-Year Median

1: Calculate the cases per day
2: Calculate the sequence of dates define from user
3: Match the cases with the complete dates and format the data
4: Sum the cases by week
5: Calculate the median weekly value from the previous three years
6: Define the anomalous activity if the weekly case counts is greater than the previous three year median weekly cumulative value

References

[1] Schneider P, Xhafa F. Chapter 3 - Anomaly detection: Concepts and methods. In: Schneider P, Xhafa F, editors. Anomaly Detection and Complex Event Processing over IoT Data Streams. Academic Press;. p. 49-66. Available from: https://www.sciencedirect.com/science/article/pii/B9780128238189000134.

[2] Mullineaux DR, Irwin G. Error and anomaly detection for intra-participant time-series data;4(1):28-35. Publisher: Taylor & Francis _eprint: https://doi.org/10.1080/23335432.2017.1348913. Available from: https://doi.org/10.1080/23335432.2017.1348913.

[3] Nekorchuk DM, Gebrehiwot T, Lake M, Awoke W, Mihretie A, Wimberly MC. Comparing malaria early detection methods in a declining transmission setting in northwestern Ethiopia;21(1):788. Available from: https://doi.org/10.1186/s12889-021-10850-5.

[4] Hay SI, Simba M, Busolo M, Noor AM, Guyatt HL, Ochola SA, et al. Defining and Detecting Malaria Epidemics in the Highlands of Western Kenya - Volume 8, Number 6—June 2002 - Emerging Infectious Diseases journal - CDC. Available from: https://wwwnc.cdc.gov/eid/article/8/6/01-0310_article.

[5] Cullen JR, Chitprarop U, Doberstyn EB, Sombatwattanangkul K. An epidemiological early warning system for malaria control in northern Thailand;62(1):107-14. Available from: https://www.ncbi.nlm.nih.gov/pmc/articles/PMC2536271/.

[6] Hyndman R. ARIMA;. Available from: https://search.r-project.org/CRAN/refmans/forecast/html/auto.arima.html.

[7] 8.9 Seasonal ARIMA models | Forecasting: Principles and Practice (2nd ed);. Available from: https://otexts.com/fpp2/seasonal-arima.html.

[8] Manso P, Fernandez J. TSclust: Time Series Clustering Utilities;. Available from: https://cran.r-project.org/web/packages/TSclust/index.html.

[9] Montero P, Vilar J. TSclust : An R Package for Time Series Clustering;62:1-43.

[10] Thang TM, Kim J. The Anomaly Detection by Using DBSCAN Clustering with Multiple Parameters. In: 2011 International Conference on Information Science and Applications;. p. 1-5. ISSN: 2162-9048. Available from: https://ieeexplore.ieee.org/document/5772437.

[11] Hahsler M. dbscan: Density-Based Spatial Clustering of Applications with Noise (DBSCAN) and Related Algorithms;. Available from: https://cran.r-project.org/web/packages/dbscan/index.html.

[12] Lertpiriyasuwat C, Sudathip P, Kitchakarn S, Areechokchai D, Naowarat S, Shah JA, et al. Implementation and success factors from Thailand's 1-3-7 surveillance strategy for malaria elimination;20(1):201. Available from: https://doi.org/10.1186/s12936-021-03740-z.

[13] Roh M, Lausatianragit K, Chaitaveep N, Jongsakul K, Sudathip P, Raseebut C, et al. Civilian-military malaria outbreak response in Thailand: an example of multi-stakeholder engagement for malaria elimination;20.

[14] Thailand Malaria Elimination Program;. Available from: https://malaria.ddc.moph.go.th/malariar10/index_newversion.php.
