# Supplementary material for: Early warning systems for malaria outbreaks in Thailand: an anomaly detection approach
Source: Malar J. 2024 Jan 8;23:11. doi: 10.1186/s12936-024-04837-x (PMC10775623; doi:10.1186/s12936-024-04837-x)
Supplement: Supplementary file 12 — Additional file 12: Detailed Descriptions of Verified Outbreaks. [file 12936_2024_4837_MOESM12_ESM.pdf]

Detailed Descriptions of Verified Outbreaks

To validate the anomaly detection algorithms, real outbreak dates were found through literature and confirmed with the online Thailand Malaria Elimination Tool and BIOPHICS. 7 malaria outbreaks in Thailand are described. The province of Ubon Ratchathani had outbreaks in 2014 and 2015 due to unregulated deforestation and lacked a functioning early warning detection and response [1]. In April 2017, an outbreak was notified in the Sisaket province most likely due to rubber tapping [2]. A province located at the Thai-Myanmar border, Tak, is one of three provinces with the highest incidence of malaria in Thailand and common to outbreaks [3]. Although there has been a general decreasing trend from 2012 to 2015, analysis of the incidence shows a large spike in cases from 2021 to 2022 [3]. Another province with a high incidence of malaria is Yala [4]. In 2016, Yala had a large increase in cases and has been an endemic area for malaria for many years due to civil unrest and conflict [4]. Another province with recent outbreaks is Kanchanaburi [4]. In 2017, an outbreak was reported in the Kradan Subdistrict and similarly, a large spike in cases was observed from 2021 to 2022 [4].

References

[1] Asia ROfSE, Organization WH. Programmatic review of the national malaria programme in Thailand: summary report. WHO Regional Office for South-East Asia;. Available from: https://apps.who.int/iris/handle/10665/253958.

[2] Roh M, Lausatianragit K, Chaitaveep N, Jongsakul K, Sudathip P, Raseebut C, et al. Civilian-military malaria outbreak response in Thailand: an example of multi-stakeholder engagement for malaria elimination;20.

[3] Mercado CEG, Lawpoolsri S, Sudathip P, Kaewkungwal J, Khamsiriwatchara A, Pan-ngum W, et al. Spatiotemporal epidemiology, environmental correlates, and demography of malaria in Tak Province, Thailand (2012–2015);18(1):240. Available from: https://doi.org/10.1186/s12936-019-2871-2.

[4] Guide to Malaria Elimination For Thailand's Local Administrative Organizations and the Health Network. Bureau of Vector Borne Diseases, Department of Disease Control, Ministry of Public Health;.
