# Supplementary material for: Early warning systems for malaria outbreaks in Thailand: an anomaly detection approach
Source: Malar J. 2024 Jan 8;23:11. doi: 10.1186/s12936-024-04837-x (PMC10775623; doi:10.1186/s12936-024-04837-x)
Supplement: Supplementary file 13 — Additional file 13: The pseudocode for validating and comparing various anomaly detection methods. [file 12936_2024_4837_MOESM13_ESM.pdf]

```
List the methods we want to compare
Extract the unique list of province names
Load the outbreak CSV file and rename the columns
Set the date range we want to conduct our analysis on
for Iterate over methods do
    for Iterate over provinces do
        Filter the data for each province
        Check if the filtered data contains records
        if the data has reads then
            Apply anomaly detection algorithm to current province data
            Record all detected anomalies
        end if
        Count anomalies alerted within 14 days prior to verified outbreak dates
        Record the province name, method used, total number of anomalies triggered, and number of verified anomalies triggered
    end for
end for
Summarise the data to show how many true anomalies were caught for each method
```
