# Supplementary material for: Early warning systems for malaria outbreaks in Thailand: an anomaly detection approach
Source: Malar J. 2024 Jan 8;23:11. doi: 10.1186/s12936-024-04837-x (PMC10775623; doi:10.1186/s12936-024-04837-x)
Supplement: Supplementary file 14 — Additional file 14: Visualisation of Thresholds for Time Series Methods. [file 12936_2024_4837_MOESM14_ESM.pdf]

Visualisation of Thresholds for Time Series Methods

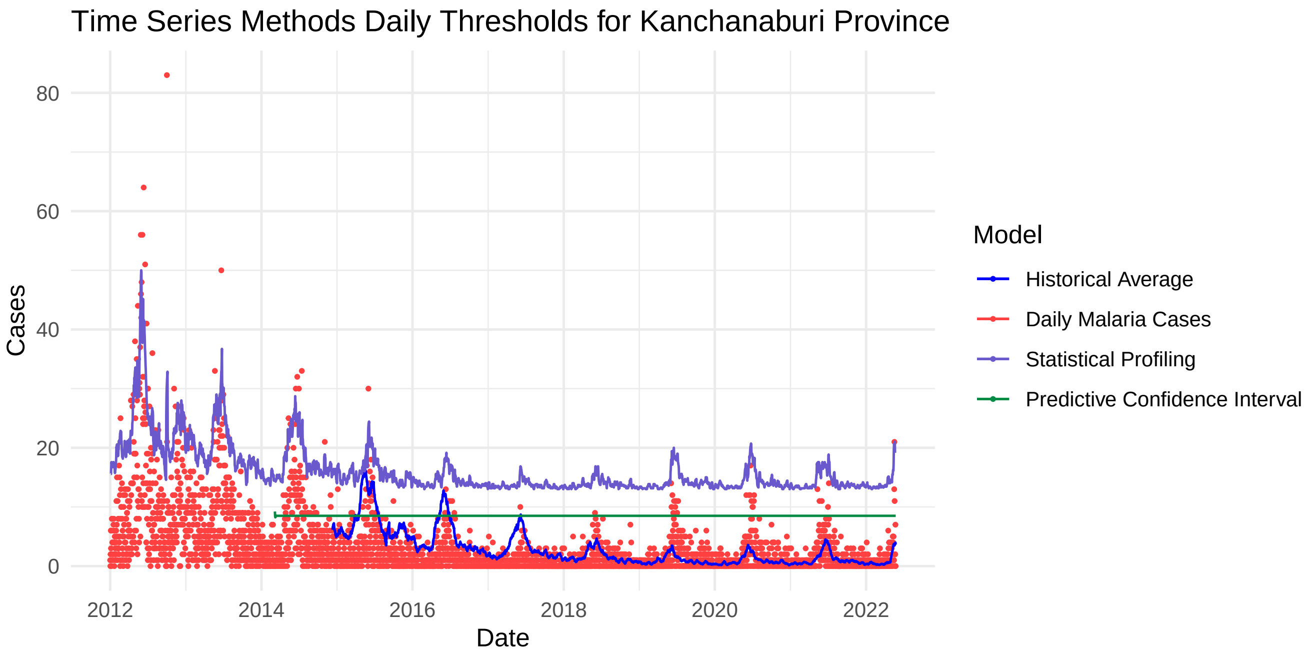

Fig. 1: Time Series Methods Using Daily Malaria Cases Applied to the Kanchanaburi Province

Figure 1 shows time series methods which use daily aggregated malaria cases for analysis and the threshold used to classify anomalous observations. The time series methods which use daily malaria cases are Historical Average, Statistical Profiling, and Predictive Confidence Interval.

Figure 2 shows time series methods which use weekly aggregated malaria cases for analysis and the threshold used to classify anomalous observations. The time series methods which use weekly malaria cases are Weekly Case Comparison and Weekly Three-Year Median.

Figure 3 shows time series methods which use monthly aggregated malaria cases for analysis and the threshold used to classify anomalous observations. The time series method which uses monthly malaria cases are Monthly Case Comparison.

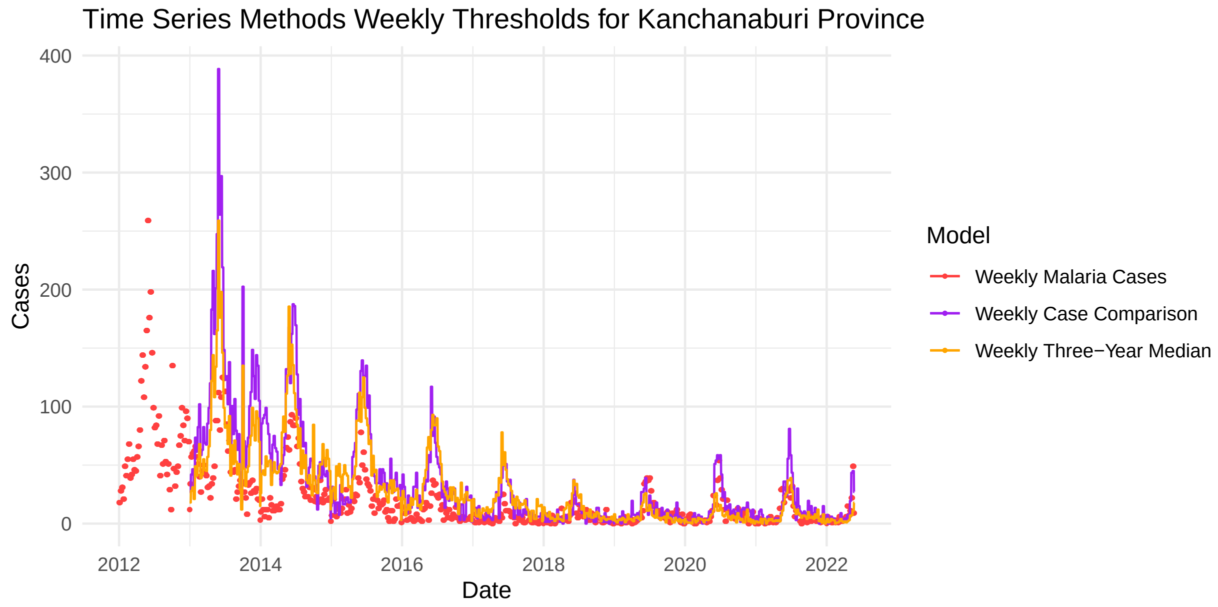

Fig. 2: Time Series Methods Using Weekly Malaria Cases Applied to the Kanchanaburi Province

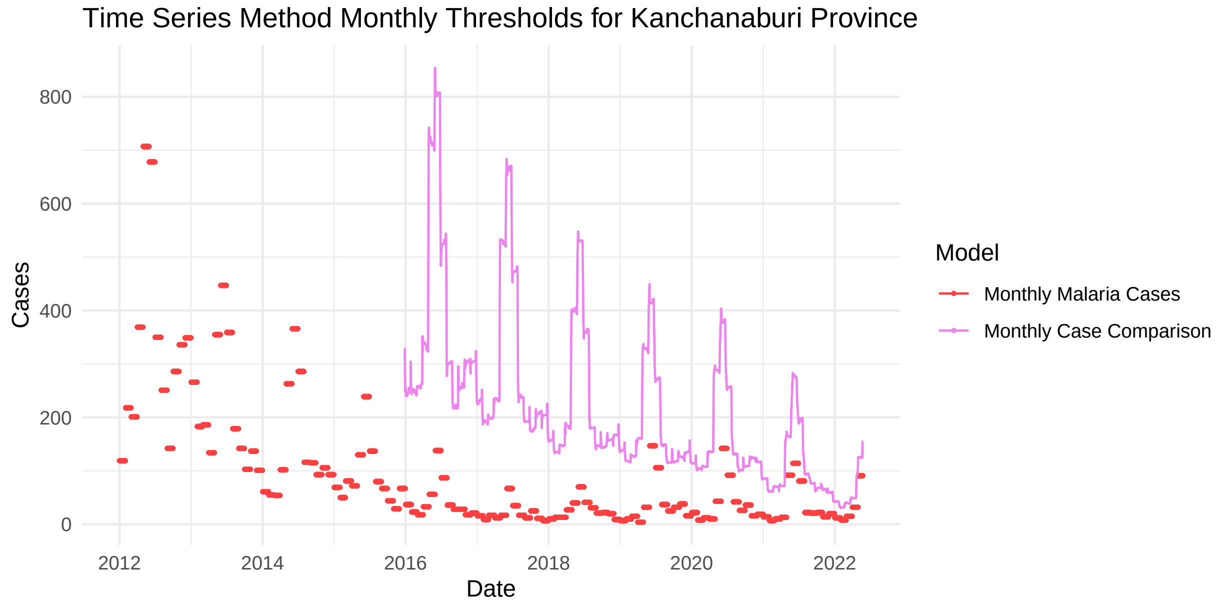

Fig. 3: Time Series Methods Using Monthly Malaria Cases Applied to the Kanchanaburi Province
